# Draft Genome Sequences of Two *Salmonella enterica* Strains Isolated from Sprouted Chia and Flax Seed Powders

**DOI:** 10.1128/genomeA.00963-16

**Published:** 2016-09-22

**Authors:** Jennifer Ronholm, Nicholas Petronella, Sandeep Tamber

**Affiliations:** a Microbiology Research Division, Bureau of Microbial Hazards, Food Directorate, Health Products and Food Branch, Health Canada, Ottawa, Ontario, Canada; b Biostatistics and Modelling Division, Bureau of Food Surveillance and Science Integration, Food Directorate, Health Products and Food Branch, Health Canada, Ottawa, Ontario, Canada

## Abstract

A 2014 foodborne salmonellosis outbreak in Canada and the United States implicated, for the first time, sprouted chia seed powder as the vehicle of transmission. Here, we report the draft whole genome sequences of two *Salmonella enterica* strains isolated from sprouted powders related to the aforementioned outbreak.

## GENOME ANNOUNCEMENT

The pathogen *Salmonella enterica* is one of the most common bacterial causes of foodborne gastroenteritis. It can survive in a variety of environments (including those with suboptimal growth conditions) and is widespread in the environment and the intestinal tracts of vertebrate animals. Consequently, many food types, including meat, produce, eggs, nuts, and seeds, can act as vehicles for *Salmonella* transmission (1–3).

In 2014, a multiprovince, multistate foodborne outbreak in Canada and the United States linked to the presence of *Salmonella* in sprouted chia seed powders occurred. During the course of the outbreak investigation, several lots of sprouted flax seed powder tested positive for the presence of *Salmonella* and were recalled from the marketplace. Multiple *Salmonella* serovars were found on these products, and this outbreak was the first to document sprouted seed powders as an agent of bacterial transmission. A project has been initiated to gain insight on the survival of *Salmonella* on sprouted seed powders (4). Here, we report the draft whole-genome sequences of two *S. enterica* strains that were isolated from sprouted chia and flax seed powders as a part of this study.

Strains were isolated according to the procedures detailed in Health Canada’s Compendium of Analytical Methods MFHPB-20 (5). Briefly, samples underwent a 24-h preenrichment in buffered peptone water at 35°C, a selective enrichment in Rappaport-Vassiliadis soya peptone broth for 24 h at 42.5°C, followed by growth on selective agar for 24 h at 35°C. Strain E3 (Biosample SAMN05356916) was isolated from bismuth sulfite agar, and strain F7 (Biosample SAMN05356917) was isolated from xylose-lysine-deoxycholate agar.

Genomic DNA was isolated from two to three colonies of a pure culture of each strain grown on tryptic soy agar using a Maxwell 16SEV cell DNA purification kit (Promega, Madison, WI, USA). Sequencing libraries were prepared with a Nextera XT DNA sample preparation kit (Illumina, San Diego, CA, USA) and read using a benchtop Illumina MiSeq instrument for 600 cycles. Reads were assembled *de novo* into high-quality draft genomes using SPAdes version 3.5.0 (6), the Mismatch Corrector tool, and BayesHammer for error correction (7). Gene predictions and annotations were performed by the NCBI Prokaryotic Genome Annotation Pipeline (PGAP) (8). The serotypes of the isolates were determined using SISTR (9) and confirmed using the traditional method according to the White-Kauffmann-Le Minor scheme (10).

The assemblies resulted in 53 (strain E3) and 36 (strain F7) nonoverlapping contiguous sequences with total lengths of 4,724,488 bp and 4,555,493 bp and sequencing coverages of 94.5-fold and 103-fold, respectively. The G+C content of each genome was 52.2%. Both the SISTR application and the traditional serotyping method revealed that strain E3 belonged to the serovar Newport and that strain F7 was a member of the Bredeney serovar.

### Accession number(s).

These sequences have been deposited in DDBJ/ENA/GenBank. The respective accession numbers for strains E3 (Biosample SAMN05356916) and F7 (Biosample SAMN05356917) are MASZ00000000 and MATA00000000. The versions described in this paper are the first versions, MASZ01000000 and MATA01000000.
